# Management decisions of an Academic Radiology Department during COVID-19 pandemic: the important support of a business analytics software

**DOI:** 10.1007/s00330-022-08709-3

**Published:** 2022-04-05

**Authors:** Andrea Laghi, Virginia Tamburi, Michela Polici, Paolo Anibaldi, Adriano Marcolongo, Damiano Caruso

**Affiliations:** 1 Department of Medical Surgical Sciences and Translational Medicine, Sapienza University of Rome - Sant’Andrea University Hospital, Via di Grottarossa, 1035-1039, 00189 Rome, Italy; 2 Department of Radiology, Federico II University Hospital, Naples, Italy; 3 Hospital Direction and Clinical Departments, Sant’Andrea University Hospital, Via di Grottarossa, 1035-1039, 00189 Rome, Italy

**Keywords:** Business analytics, Radiology, Management, COVID-19

## Abstract

**Objectives:**

To analyze the response in the management of both radiological emergencies and continuity of care in oncologic/fragile patients of a radiology department of Sant’Andrea Academic Hospital in Rome supported by a dedicated business analytics software during the COVID-19 pandemic.

**Methods:**

Imaging volumes and workflows for 2019 and 2020 were analyzed. Information was collected from the hospital data warehouse and evaluated using a business analytics software, aggregated both per week and per quarter, stratified by patient service location (emergency department, inpatients, outpatients) and imaging modality. For emergency radiology subunit, radiologist workload, machine workload, and turnaround times (TATs) were also analyzed.

**Results:**

Total imaging volume in 2020 decreased by 21.5% compared to that in 2019 (*p* < .001); CT in outpatients increased by 11.7% (*p *< .005). Median global TAT and median code-blue global TAT were not statistically significantly different between 2019 and 2020 and between the first and the second pandemic waves in 2020 (all *p *> .09). Radiologist workload decreased by 24.7% (*p *< .001) during the first pandemic wave in 2020 compared with the same weeks of 2019 and showed no statistically significant difference during the second pandemic wave, compared with the same weeks of 2019 (*p* = 0.19).

**Conclusions:**

Despite the reduction of total imaging volume due to the COVID-19 pandemic in 2020 compared to 2019, management decisions supported by a dedicated business analytics software allowed to increase the number of CT in fragile/oncologic outpatients without significantly affecting emergency radiology TATs, and emergency radiologist workload.

**Key Points:**

*• During the COVID-19 pandemic, management decisions supported by business analytics software guaranteed efficiency of emergency and preservation of fragile/oncologic patient continuity of care.*

*• Real-time data monitoring using business analytics software is essential for appropriate management decisions in a department of radiology.*

*• Business analytics should be gradually introduced in all healthcare institutions to identify strong and weak points in workflow taking correct decisions.*

**Supplementary Information:**

The online version contains supplementary material available at 10.1007/s00330-022-08709-3.

## Introduction

The international outbreak of novel SARS-CoV-2 (severe acute respiratory syndrome coronavirus 2) [1, 2] and the associated coronavirus disease 2019 (COVID-19) had crushing effects both on the global population and on healthcare systems due to its high transmissibility, elevated mortality among elderly and fragile patients, and lack of effective therapy [3]. In particular, the need for intensive care for many patients has put severe pressure on hospitals, whose emergency departments (EDs) and intensive care units (ICUs) were unprepared [4, 5]. Moreover, since COVID-19’s presentation is often an interstitial pneumonia, whose diagnosis and clinical evaluation rely heavily on imaging findings, radiology units have been at the forefront of the emergency, with a huge number of patients requiring chest imaging, either X-ray or high-resolution CT [6–8].

The pandemic brought up two kinds of problems for radiologists: on one hand, the diagnostic aspects that required specific training given the peculiarities of X-ray and CT findings of COVID-19 pneumonia [9], and on the other hand, the unprecedented complex organizational problems [10]. Radiology departments were asked to completely reorganize the workload in a matter of a few days, by taking critical decisions regarding the management of acute emergencies as well as those of chronic oncologic and fragile patients.

Main urgent needs were (1) creating separate paths for COVID-19 and non-COVID-19 patients, to avoid cross-contamination of patients; (2) integrating a dedicated CT scanner in the COVID-19 path to improve diagnostic accuracy; (3) intensifying personnel (radiologists and radiographers) at the ED, particularly for night shifts; and (4) guaranteeing diagnostic exams in frail and cancer patients [10, 11]. Putting these organizational changes into practice in a sudden and precise way was extremely complex and particularly true for healthcare organizations who lack real-time information on workflows and on modifications brought on by unexpected external events, such as, in this case, the COVID-19 outbreak. A solution might be the implementation of a business analytics (BA) tool to support management decisions [12]. BA refers to the procedural and technical infrastructure that collects, stores, and analyzes data produced by a company’s activity by providing metrics and graphical dashboards for an objective real-time measurement of business efficiency [12].

In this study, we reported the response of a radiology department of an academic hospital during the two pandemic waves that occurred in Rome from March 9 to April 12 (weeks 11–15) and from November 2 to November 29, 2020 (weeks 45–48) [14]. Moreover, we examined the department governance choices guided by the support of a dedicated BA software, having the management of emergencies and the guarantee of continuity of care, especially for oncologic and fragile patients, as the two main objectives.

## Materials and methods

### Study design

A retrospective review of the imaging volumes and workflows of the radiology unit of Sant’Andrea Academic Hospital in Rome for 2019 and 2020 was performed. The institutional ethical committee approved the study (ref. nr CE 5773_2020).

Our institution is a medium-size academic hospital; it counts 452 beds, 7 departments with 48 units, 523 doctors, and 1063 healthcare professionals. It is a center of excellence for thoracic surgery, medical oncology, and orthopedic surgery. Radiology unit includes three subunits: interventional radiology (IR); breast imaging and general radiology; and emergency radiology, located in a different floor of the hospital, embedded in the ED, which belongs to general radiology. Radiology personnel is made of 23 radiologists (4 dedicated to IR), 38 residents, 41 radiographers, and 15 nurses. Three digital radiology (DR) units, two interventional radiology (IR) suites, two multidetector computed tomography (CT) scanners, two 1.5-T magnetic resonance (MR) scanners, one MSK-dedicated low-field MR scanner, one ultrasound scanner (US), one mammography equipment, and one bone densitometry scanner (DXA) are available in the main department. The emergency radiology unit is fully equipped with DR, US, and MDCT scanners. The same scanners were available in 2019 and 2020 and no major changes in radiology personnel occurred.

With the COVID-19 outbreak, our hospital was selected by the Regional Healthcare System as one of the nine hubs in our region. As a result, dedicated COVID-19 beds were opened in intensive care unit (ICU) (nr, 32) and high-intensity (nr, 40) and low-intensity (nr, 76) care units.

### Data collection and analysis

Data were collected from the hospital data warehouse (electronic records, RIS, and PACS data from 2015 onwards) and evaluated using a data analytics and business intelligence software operating since March 2019 (Radiology Command Center™, GE Healthcare), which provides real-time, actionable information accessible by staff and hospital leaders. Data from years 2019 and 2020 were aggregated both per quarter and per week during the selected time intervals, stratified by patient service location (emergency, inpatients, outpatients) and by imaging modality (DR, mammography, DXA, ultrasound, CT, MRI, and interventional radiology).

For the emergency radiology only, we collected metrics of the average number of examinations per hour, CT exam allocation between primary CT scanner (CT2) and secondary/backup CT scanner (CT1), and median turnaround time (TAT). TAT was defined as the time from the request of the radiological examination from the emergency physicians to the password-secured electronic signature of the radiological report from a radiologist. Turnaround time was analyzed considering all radiological exams (global TAT), code-blue only patients (code-blue global TAT), CT examinations only (CT TAT), and CT performed in code-blue patients (code-blue CT TAT).

Data obtained by our data analytics and business intelligence software were exported to Microsoft Excel and descriptive statistics were performed to assess the number of patients and imaging exams in 2019 and 2020, divided by quarters. The total number and relative percentage of imaging exams (DR, mammography, DXA, US, CT, MRI, and IR) and patients, stratified as emergency, inpatients, and outpatients, were calculated, and evaluated.

### Statistical analysis

All analyses and graphs were performed using commercially available software SPSS (IBM Corp. Released 2017. IBM SPSS Statistics for Macintosh, Version 25.0.: IBM Corp.). Quantitative variables have been expressed as mean ± standard deviations; categorical variables have been conveyed as frequencies and percentages. The Kolmogorov-Smirnov test was used to assess data distribution. In case of Gaussian distribution, data were tested with Student’s *t* test, while the Wilcoxon test was applied for non-Gaussian distributed data. Percentages were compared by using the Pearson’s chi-square test or in alternative Fisher’s exact test. A *p* < 0.05 was considered to indicate a statistically significant difference.

## Results

**Fig. 1:**
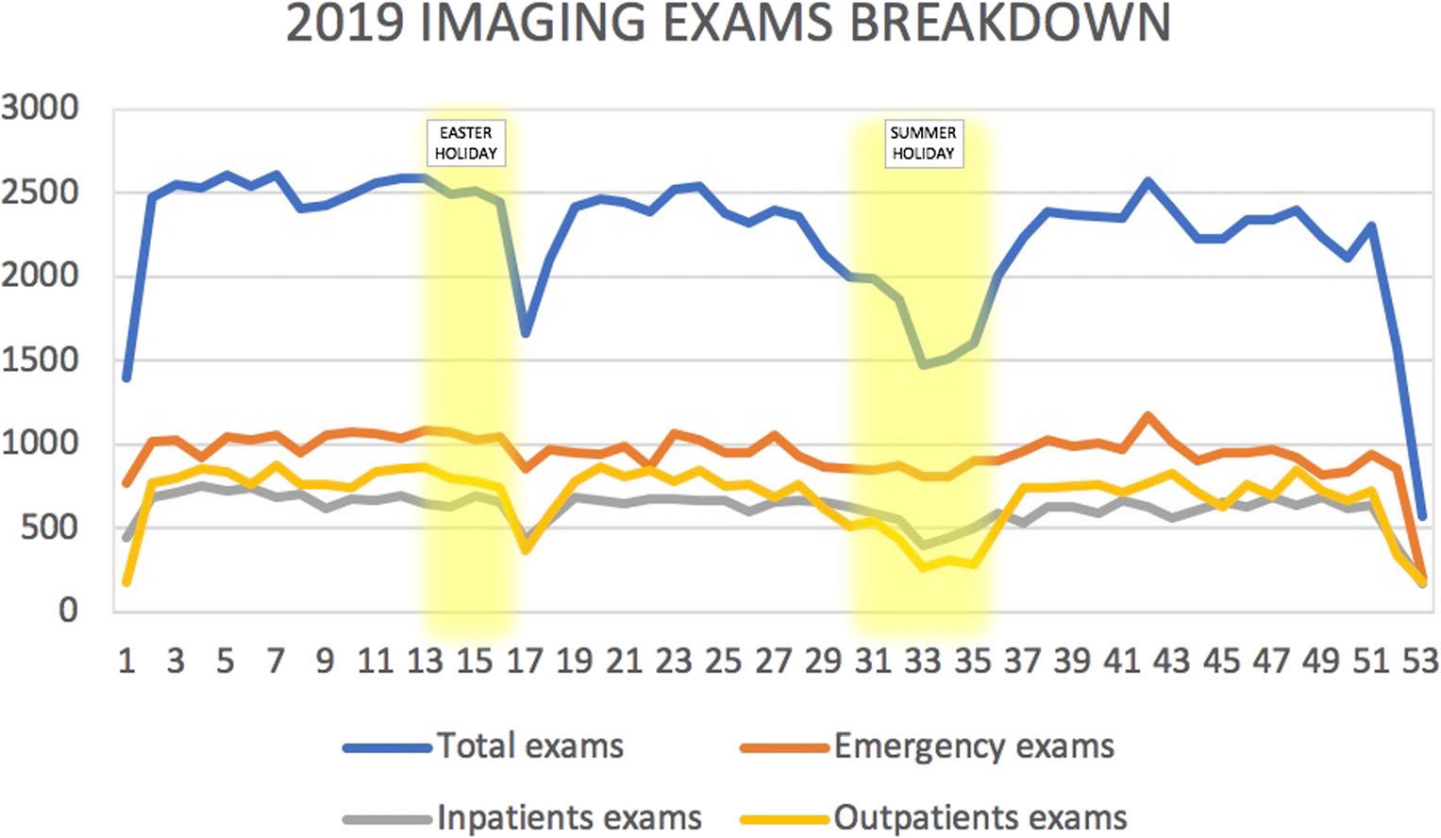
Graphical representation of 2019 exams’ trend, stratified for total, emergency, inpatients, and outpatients

**Fig. 2:**
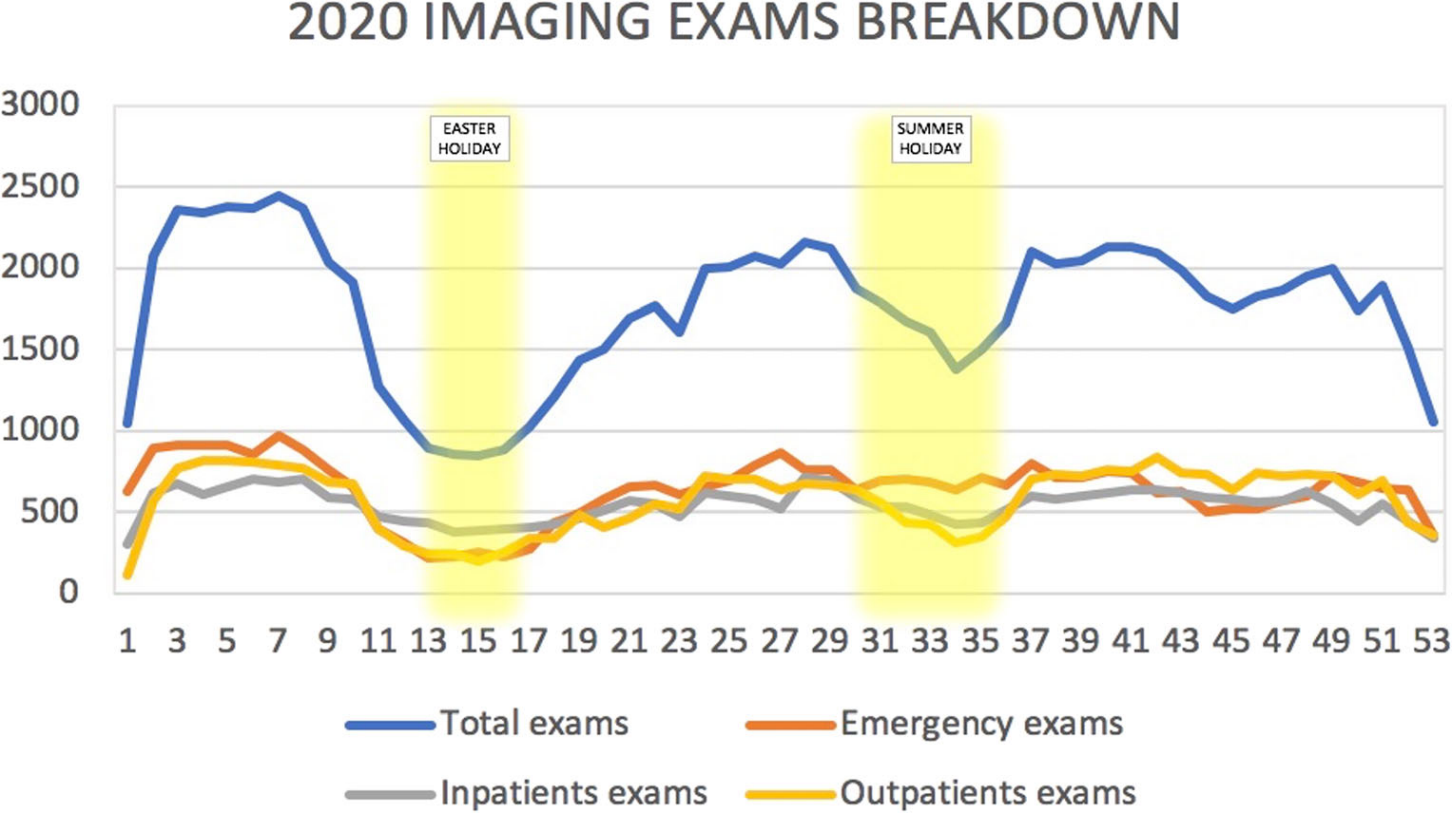
Graphical representation of 2020 exams’ trend, stratified for total, emergency, inpatients, and outpatients

Total imaging volume in 2020 marked a 21.5% decrease compared to 2019 (93,292 examinations in 31,626 patients in 2020 vs. 118,901 examinations in 43,635 patients in 2019; *p *< .001). When analyzed quarterly, there was a decrease of − 22.7% examinations in the 1st Qrt (22,661 in 2020 vs. 31,804 in 2019, *p *< .001), − 38.3% in the 2nd Qrt (18,096 in 2020 vs. 30,710 in 2019, *p *< .001), − 9% in the 3rd Qrt (23,976 in 2020 vs. 26,355 in 2019, *p *< .001), and − 14.1% in the 4th Qrt (25,783 in 2020 vs. 30,032 in 2019, *p *< .001). Detailed weekly volume analyses of 2019 and 2020 for the three different types of patients (emergency, inpatients, and outpatients) are available in Figs. 1 and 2, respectively.

Considering single imaging modalities, a reduction of DR (− 32.6%; 61,462 in 2019 vs. 41,408 in 2020, *p *< .001), mammography (− 7.8%; from 2059 to 1899, *p* .011), DXA (− 24%; from 2478 to 1883, *p *< .001), US (− 27.7%; from 17,213 to 12,440, *p *< .001), MRI (− 25.1%; from 3179 to 2380, *p* = .003), and IR (− 20.4%; from 2440 to 1942, *p *< .001) was observed. CT examinations increased by 2.3% (from 32,730 to 33,485, *p* = .34) (Table 1).

**Table 1:** Comparison between imaging examinations performed in 2019 and 2020

	2019	2020	%	p
Total	118,901	93,292	− 21.54%	< .001
Emergency	50,252	33,798	− 32.74%	< .001
Inpatients	32,658	28,883	− 11.56%	< .001
Outpatients	36139	30,611	− 15.3%	< .001
DR	61,462	41,408	− 32.63%	< .001
Mammography	2059	1899	− 7.77%	.011
DXA	2478	1883	− 24.01%	< .001
US	17,213	12,440	− 27.73%	< .001
CT	32,730	33,485	+ 2.31%	.34
MRI	3179	2380	− 25.13%	.003
IR	2440	1942	− 20.41%	< .001

*DR*, digital radiology; *US*, ultrasound sonography; *CT*, computed tomography; *MRI*, magnetic resonance; *IR*, interventional radiology

Considering hospital admission (emergency, inpatients, and outpatients), during the first pandemic wave (weeks 11–15) the largest drop in examinations was observed for emergency patients (5295 examinations in 2020 vs. 1420 in 2019, − 73.2%, *p *< .001), followed by outpatients (1387 examinations in 2020 vs. 4142 in 2019, − 66.4%, *p *< .001) and inpatients (2178 examinations in 2020 vs. 3372 in 2019, − 35.4%, *p *< .001); during the second pandemic wave (weeks 45–48), the only significant reduction of examinations was registered for emergency patients (2213 examinations in 2020 vs. 3791 in 2019, − 41.7%, *p *< .001), and for inpatients (2342 inpatient examinations in 2020 vs. 2604 in 2019, − 10.1%, *p *< .001). Outpatients’ data showed no significant decrease in the number of examinations (2838 outpatient examinations in 2020 vs. 2923 in 2019, − 3.0%, *p* = .12) (Tables 2 and 3). If considering CT in outpatients, 10231 examinations were performed in 2020 compared with 9030 examinations in 2019 (+ 11.7%; *p *< .005). Per quarter, a decrease of − 35% (1540 examinations in 2020 vs. 2380 in 2019, *p *< .001) and of − 25% (1590 in 2020 and 2120 in 2019, *p *< .001) were registered in the 1st and 2nd quarters respectively and an increase of + 16% (1680 examinations in 2020 vs. 1410 examinations in 2019, *p *< .001) and of + 10.6% (1790 in 2020 vs. 1590 in 2019, *p *< .001) were registered in the 3rd and 4th quarters respectively.

**Table 2:** Deep analysis of emergency patients during the first (11–15 weeks) and second (45–48 weeks) pandemic peaks

	11–15 weeks				45–48 weeks				2019	2020
	*2019*	*2020*	*%*	*p*	*2019*	*2020*	*%*	*p*		
Emergenc**y**	5295	1420	− 73.2%	< .001	3791	2213	− 41.7%	< .001		
Waiting time	15.8 min	20.5 min	+ 23%	.34	14.9 min	15.8 min	+ 6%	.85	15.1 min	14.9 min
Avg N exams per hour	3.89	2.93	− 24.7%	< .001	3.36	3.48	+ 3.4%	1.00	4.96	3.98
CT	1580	1030	− 34.8%	< .001	1391	1281	− 7.8%	.03	32730	33485
Waiting time	18 min	26.8 min	+ 48.8%	.17	17.7 min	18.7 min	+ 5.6%	.85	18.3 min	18.9 min
Thorax	196	639	+ 226%	< .001	197	646	+ 136%	< .001		
Abdominal	376	127	− 66.2%	< .001	328	204	− 37.8%	< .001		
Head	616	165	− 73.2%	< .001	482	227	− 42.6%	< .001		
DR	3330	364	− 89.1%	< .001	2094	848	− 59.5%	< .001	61462	41408
US	372	21	− 94.4%	< .001	281	54	− 80.8%	< .001	17213	12440

*CT*, computed tomography; *DR*, digital radiology; *US*, ultrasound sonography

**Table 3:** Deep analysis of inpatients during the first (11–15 weeks) and second (45–48 weeks) pandemic peaks

	11–15 weeks				45–48 weeks			
Examinations	*2019*	*2020*	*%*	*p*	*2019*	*2020*	*%*	*p*
Total inpatients	3372	2178	− 35.4%	< .001	2604	2342	− 10.1%	< .001
CT	683	554	− 18%	< .001	567	606	+ 6.8%	.254
Thorax	240	273	+ 13.7%	.145	192	264	+ 37.5%	< .001
Abdominal	207	130	− 37.2%	< .001	168	137	− 18.5%	.07
DR	2150	1370	− 47.6%	< .001	1684	1439	− 14.6%	< .001
US	250	131	− 94.4%	< .001	151	134	− 11.1%	.313
MRI	29	27	− 6.9%	.789	39	36	− 7.7%	.729
IR	260	96	− 63.1%	< .001	173	150	− 13.3%	.20
Total outpatients	4142	1387	− 66.4%	< .001	2923	2838	− 3.0%	.12
CT	1119	555	− 50%	< .001	652	1099	+ 41%	< .001
CR	1146	305	− 73%	< .001	852	605	− 29%	< .001
US	1232	463	− 62%	< .001	1074	769	− 28%	< .001
MRI	342	43	− 87%	< .001	250	186	− 26%	.002
IR	4	3	− 25%	.705	7	5	− 29%	.56
Mammography	207	66	− 68%	< .001	179	214	+ 16%	.07
DXA	299	18	− 94%	< .001	88	174	+ 49%	< .001

*CT*, computed tomography; *DR*, digital radiology; *US*, ultrasound sonography; *MRI*, magnetic resonance; *IR*, interventional radiology; *DXA*, bone densitometry

### Turnaround time metrics

Comparing 2019 and 2020, median global TATs were 59 min in 2019 vs. 65 min in 2020 (*p* = .11); median code-blue global TATs were 65 min and 74 min, respectively (*p* = .09); median CT TATs were 81 min and 91 min, respectively (*p* = .04); and median code-blue CT TATs were 75 min and 80 min, respectively (*p* = .11) (Table 4).

**Table 4:** Deep analysis of median turnaround time in 2019 and in 2020

	2019	2019 (weeks 11–15)	2019 (weeks 45–48)	2020	2020 (weeks 11–15)	2020 (weeks 45–48)
Total CT exams	17,005	1532	1371	16,290	1002	1277
CT 1 (backup)	2631 (15.5%)	137 (8.9%)	15 (1.1%)	2256 (13.9%)	226 (22.6%)	127 (9.9%)
CT 2	14374	1395	1356	14034	776	1150
Median TAT, all exams	59 min	53 min	65 min	65 min	92 min	75 min
Median TAT CT	81 min	75 min	81 min	91 min	109 min	107 min
Median TAT, all exams, code-blue patients	65 min	59 min	64 min	74 min	81 min	85 min
Median TAT CT, code-blue patients	75 min	65 min	69 min	80 min	91 min	83 min

*CT*, computed tomography; *TAT*, turnaround time

Considering 2020, during the first (weeks 11–15) and the second (weeks 45–48) pandemic waves, median global TATs were 92 min and 75 min (*p* = .09); median code-blue global TATs were 81 min and 85 min, respectively (*p* = .16); median CT TATs were 109 min and 107 min, respectively (*p* = .34); and median code-blue CT TATs were 91 min and 83 min, respectively (*p* = .09).

### Radiologist workload

During the first pandemic peak (weeks 11–15, 2020), a 24.7% decrease in the radiologist workload was registered compared with the same period of 2019: 2.93 exams/h in 2020 vs. 3.89 exams/h in 2019 (*p *< .001). During the second pandemic peak (weeks 45–48), no statistically significant difference was registered: 3.48 exams/h in 2020 vs. 3.36 exams/h in 2019; + 3.4% (*p* = 1.0) (Table 2).

### Backup CT

On backup CT (CT1), in 2020, 2256 out of 16,290 (13.9%) emergency CT examinations were performed in comparison with 2019, when emergency CT examinations were 2631 out of 17,005 (15.5%) (*p* = .02). Considering 2020, 226 out of 1002 (22.6%) and 127 out of 1277 (9.9%) emergency CT examinations were performed during the two pandemic waves (weeks 11–15 and weeks 45–48) respectively.

## Discussion

Our results demonstrate that, despite the pandemic and the decision of our regional healthcare system to include Sant’Andrea Academic Hospital among the nine regional COVID-19 hubs, and despite the significant reduction of about one-fifth of the total radiological examinations, our radiology unit was able to increase by around 12% the number of CT examinations for outpatients, particularly fragile and oncologic patients, in 2020 compared to 2019.

This result was the consequence of the different decisions about the opening and closure of radiological paths for COVID-19 and non-COVID-19 patients, taken during the first and the second pandemic peaks and driven by the availability of real-time data analytics about number and distribution of diagnostic examinations. However, caution is needed when interpreting our data, because the epidemiology of the pandemic in Rome and the role of our hospital within the regional healthcare network may have affected our results.

The pandemic in Rome came in two waves: the first one in March–April 2020 (weeks 11–15) and the second one in November 2020 (weeks 45–48).

At the beginning, alarming media reports regarding Rome together with a dramatic increase in COVID-19 patients admitted to the ED suggested to take the decision to separate the emergency diagnostic paths for COVID-19 and non-COVID-19 patients, to avoid cross-contamination. The radiology subunit embedded in the ED became dedicated to COVID-19 patients and had its own emergency radiology team, while a second emergency path for non-COVID-19 patients was set up in the main radiology department, on a different floor, including one of the two CT scanners usually available for in-/outpatients. The reason behind such logistic choice was mostly an emotional response to an unprecedented alert situation that called for extreme health safety measures, certainly not justified by either number of examinations or radiologist workload, at least in our hospital. In fact, number of examinations showed a progressive reduction starting from week 8 (February 17, 2020) when the government confirmed the first twenty cases and reported the first death, with a clear downwards trendline. On the 11th week (when on March 9, 2020, the national lockdown began), there was a 41% drop of outpatient examinations, compared to the 10th week, and the progressive decline of outpatient examinations, including CT for fragile and oncologic patients, continued until the 15th week.

Reasons for patients’ behavior were probably the fear of contagion in the hospital environment and the cancelation of most visits and non-urgent surgeries imposed by the government. Probably, the decrease of CT for fragile and oncologic outpatients was also, at least in part, the consequence of the reduction of available slots due to the re-organization of emergency radiology paths. In fact, with the improvement of the pandemic, at the time of partial reopening of the national governmental lockdown (May 4th, beginning of week 19), the standard organization (emergency radiology for both COVID-19/non-COVID-19 emergency patients and main department devoted to in-/outpatients) was re-established and the number of outpatient examinations started recovering.

At the time of the second pandemic wave (weeks 45–48), data analysis of the previous three quarters of the year suggested the possibility to maintain the standard organization. In fact, the separation of the emergency paths in two different floors of the hospital was not justified by workload changes because an in-depth analysis of the emergency radiology during the first pandemic wave showed an average reporting of 2.93 exams/h, around 25% lower than 2019 (3.89 exams/h). Emergency radiology workload not only decreased, but it became less complex with a more than three-fold increase in chest CT scans and a reduction of more than 70% of brain CT and more than 65% of abdominal-pelvic CT. These data are in line with what were reported among Italian stroke units [13] and surgical [14], orthopedic, and traumatology [15] EDs, where a dramatic decrease of hospitalization was observed.

A possible criticism at the decision of keeping a single path in the emergency radiology setup sharing CT for COVID-19 and non-COVID-19 patients might be related to difficult sanitation procedures and possible overlapping of urgent cases. In our experience, we established stringent internal CT protocols, allowing scans of COVID-19 patients consecutively and safely while sanitizing the CT scanner room at the end of the shift and not after each single CT examination [16]. Nevertheless, we had continuously monitored ER accesses to be alerting by any critical scenarios weekly, ensuring the right assistance for acute patients and avoiding any unjustified delays in diagnosis. Registered TATs confirmed our choice: overall median CT TAT in 2020 was 10 min (+ 11%) higher than in 2019 and median CT TAT in code-blue patients was 9 min (+ 13.8%) higher than in 2019. The overall increasing of TAT in 2020 could be explained by the procedures of sanitization after each shift of COVID-19 patients and by clinical condition of COVID-19 patients, who were often critical, needing a longer preparation time in the CT scanning room by the radiographers, nurses, and ICU doctors.

Despite the higher number of ED patients admitted in November (nr 354) compared to those admitted in March–April (354 vs. 294; + 16.9%), CT TAT and CT TAT in code-blue patients during weeks 45–48 were not statistically different from those weeks 11–15.

Emergency radiology workload slightly increased (3.48 exams/h) compared to the first peak (+ 16%), but it was still well below the standards of 2019 (3.89 exams/h), thus justifying our correct choice of keeping personnel allocation unchanged.

The risk of a simultaneous urgent case was balanced by the availability of a second backup CT in the main department, whose use did not affect the overall CT productivity.

Finally, the decision to keep the main department completely COVID-19 free, with both CT scanners available for in- and outpatients, allowed us to increase by + 12% the number of CT for outpatients in the 4th quarter.

In conclusions, in times of crisis, resilience is a fundamental quality to possess. It allows for change to happen rapidly and smoothly. Business analytics software is an extremely powerful tool that provides a precise insight of real-time changing scenarios. When applied to our radiology unit, it allowed us to reinforce managerial decisions and to support the changes in workforce planning during pandemic crisis, in which the main hospital goals were management of emergencies and the guarantee of continuity of care, especially for oncologic and fragile patients.

## Supplementary information

ESM 1 (DOCX 17 kb)

## Abbreviations

BA: Business analytics
DR: Digital radiology
DXA: Bone densitometry
ED: Emergency department
ICU: Intensive care unit
IR: Interventional radiology
PACS: Picture archiving and communication systems
RIS: Radiology information systems
TAT: Turnaround time

## Declarations

### Guarantor

The scientific guarantor of this publication is Prof. Andrea Laghi, MD.

### Statistics and biometry

The first and last authors have significant statistical expertise, despite no complex statistical methods were necessary for this paper.

### Methodology

• retrospective

• observational

• performed at one institution
