# Supplementary material for: Management decisions of an Academic Radiology Department during COVID-19 pandemic: the important support of a business analytics software
Source: Eur Radiol. 2022 Apr 5;32(10):7048–55. doi: 10.1007/s00330-022-08709-3 (PMC8981182; doi:10.1007/s00330-022-08709-3)
Supplement: Supplementary file 1 — (DOCX 17 kb) [file 330_2022_8709_MOESM1_ESM.docx]

**Supplementary Material**

**RADIOLOGICAL EQUIPMENT AVAILABLE IN THE RADIOLOGY UNIT**

**Main Department**

- Digital radiology units: Opera D4000RAD DR Digital System, GMM; HirisRf43 Mobile X-Rays units, Mecall GMM; CS-7 V1.35R00_014 X-Rays, Konica Minolta Inc.
- IR suites: Innova 2000 x-ray angiography system, GE Healthcare, and OEC 9800 Plus C-Arm System, GE Healthcare
- CT: Revolution EVO, GE Healthcare and Brilliance iCT 256 slice, Philips Healthcare
- MR: Magnetom AERA, Siemens Healthineers and O-Scan, Esaote
- US: MyLab ^TM^ X6, Esaote

**Emergency Radiology**

- Digital X-ray: EIDOS 3000 DR Digital Radiography, MECALL GMM
- US: MyLab ^TM^ Twice, Esaote
- CT: GE Revolution EVO, GE Healthcare.
